# The application of functional imaging in visual field defects: a brief review

**DOI:** 10.3389/fneur.2024.1333021

**Published:** 2024-02-12

**Authors:** Wangxinjun Cheng, Jingshuang Liu, Tianqi Jiang, Moyi Li

**Affiliations:** ^1^Department of Rehabilitation, The First Affiliated Hospital of Nanchang University, Nanchang, China; ^2^Queen Mary College, Nanchang University, Nanchang, China; ^3^The First Clinical Medical College, Nanchang University, Nanchang, China

**Keywords:** functional imaging, visual field defects, MRI, fMRI, OCT, DTI, OCTA

## Abstract

Visual field defects (VFDs) represent a prevalent complication stemming from neurological and ophthalmic conditions. A range of factors, including tumors, brain surgery, glaucoma, and other disorders, can induce varying degrees of VFDs, significantly impacting patients’ quality of life. Over recent decades, functional imaging has emerged as a pivotal field, employing imaging technology to illustrate functional changes within tissues and organs. As functional imaging continues to advance, its integration into various clinical aspects of VFDs has substantially enhanced the diagnostic, therapeutic, and management capabilities of healthcare professionals. Notably, prominent imaging techniques such as DTI, OCT, and MRI have garnered widespread adoption, yet they possess unique applications and considerations. This comprehensive review aims to meticulously examine the application and evolution of functional imaging in the context of VFDs. Our objective is to furnish neurologists and ophthalmologists with a systematic and comprehensive comprehension of this critical subject matter.

## 1 Introduction

Visual field defects (VFDs) denote impairments within the visual field. Umerous diseases have the potential to impact the visual field, such as retinal detachment (1), glaucoma (2, 3), retinal vein obstruction (4, 5), retinal pigmentary degeneration (6, 7), and brain tumors (8, 9) or cerebrovascular disorders (10). The majority of these defects result from the infiltration of adjacent tissue by diseases, with tumor compression being the most prevalent causative factor (11, 12). Using glaucoma as an example, it constituted a significant portion of global blindness in 2010, affecting 2.1 million individuals, which accounted for 6.5% of the 34 million blind people worldwide. Additionally, out of the 191 million individuals with visual impairments globally, 4.2 million (2.2%) experienced visual impairment due to glaucoma. Projections indicate that the prevalence of glaucoma is anticipated to rise to 112 million cases by the year 2040. It’s noteworthy that alongside the ongoing pandemic, the novel coronavirus has also been explored for potential associations with VFDs (13). VFDs are increasingly emerging as a pressing concern (14).

The traditional method for diagnosing VFDs primarily depends on visual field measurement techniques. These techniques encompass two major technologies, dynamic and static, which are sometimes used in combination. However, it’s important to recognize that visual field measurement has its constraints, as it can only identify the underlying pathological factors responsible for vision impairment. Visual measurement methods are typically employed for initial assessments and preliminary evaluations (14, 15).

However, within the realm of contemporary science, a multitude of researchers are diligently exploring inventive avenues to enhance the diagnosis, treatment, monitoring, and prognosis of VFDs. Among these initiatives, the incorporation of cutting-edge technology, notably functional imaging, has risen to the forefront as a prominent focal point (16, 17).

The progress in medical imaging has introduced a multitude of innovative techniques that have gained broad acceptance and utility in clinical settings. These encompass technologies like functional magnetic resonance imaging (fMRI), single photon emission computed tomography (SPECT), positron emission computed tomography (PET), among others. These state-of-the-art methodologies play a pivotal role in visualizing anatomical structures, evaluating the functionality and metabolic processes of human tissues and organs, and unveiling molecular markers (18, 19). Functional imaging, a significant branch of medical imaging, is closely associated with neuroimaging and places a greater emphasis on the exploration of brain function mechanisms (20). As an illustration, functional brain imaging, utilizing techniques like positron emission tomography and magnetic resonance imaging, creates maps that highlight regional changes in brain activity. This approach offers a distinctive metabolic perspective on the inherent functioning of brain systems (21). Given the close connection between the causes of VFDs and the brain, functional imaging plays a significant role in this field (17, 22). Hence, we conduct a comprehensive review of the role of functional imaging throughout the entire clinical course of VFDs, along with the latest breakthroughs, to offer researchers a clear and comprehensive perspective.

## 2 Materials and methods

### 2.1 Textual analysis

A full-text search on PubMed was conducted using “visual field defect” and “functional imaging” as keywords. Based on the article types, clinical trials and randomized controlled trials were chosen for examination, resulting in a total of 74 search results. Out of these, 26 studies were identified as having strong associations with VFDs and functional imaging. The screening criteria encompassed various factors, including but not limited to:

1. The utilization of functional imaging technology in the examination and diagnosis of VFDs.
2. The application of functional imaging technology in supporting the treatment of VFDs.
3. Using functional imaging technology to predict the future prognosis of VFDs.

Upon selecting these relevant studies, it was observed that while numerous papers discussed the connection between VFDs and functional imaging, there were no review papers specifically addressing the application of functional imaging in the context of VFDs. Therefore, this review, building upon the outlined criteria and with an eye on current research trends, conducts a detailed analysis of the 26 research articles.

### 2.2 Bibliometric analysis

Our data are from the Web of Science Core collection (23), which is the foremost database integration platform for accessing global academic information. Our indexing strategy is outlined as follows: TS = (“Visual field defect “or” Visual field defects “) AND (“functional imaging “or” MRI “or” fMRI “or” OCT “or” DWI “or” DSI “or” DTI “or” OCTA “or” SD-OCT “or” iMRI “or” DTIT “or” Diffusion tensor“). We included articles and reviews while excluding other document types such as conference papers and books. Additionally, for the sake of convenience and ensuring the reliability of subsequent statistical analyses, we filtered out non-English literature. We downloaded the data and imported it into bibliometrix (24) for bibliometric analysis, focusing on the hot keywords in this field.

## 3 Results

Following the analysis and organization of the articles, we found that neurological disorders and ophthalmological diseases (particularly glaucoma), are the most common causes of VFD from a prevalence standpoint (25). Clinically, these two diseases are also frequently encountered by physicians. Therefore, in order to enhance the practical value of this article for clinical doctors, we employed the two primary causes of VFDs– neurological diseases and ophthalmic diseases – as our classification criteria. We meticulously categorized the diagnosis and utilization of functional imaging technology for different diseases within these two classifications.

### 3.1 Diseases of nervous system

The primary principle underlying VFDs induced by neurological diseases is often associated with defects or compression of the optic nerve. Neurological conditions, including epilepsy surgery, brain tumors, and post-stroke events, frequently have an impact on patients’ visual fields, resulting in VFDs (26–28). Numerous researchers have made efforts to employ functional imaging diagnostic technology to facilitate the supplementary diagnosis and treatment of VFDs stemming from nervous system diseases.

#### 3.1.1 OCT and its associated functional imaging

Optical Coherence Tomography (OCT) is considered to be a valuable tool in assisting patients with VFDs. OCT merges the power of light and computer image processing, offering micron-level resolution and millimeter-level depth of penetration. This technology allows for cross-sectional scanning of the retina, providing detailed tomography images that aid in the assessment and diagnosis of visual field issues (29–31). In recent years, OCT has made significant advancements and found widespread application in ophthalmology-related assessments. For instance, Suh et al. explored the relationship between MRI and OCT, utilizing parameters such as macular ganglion cell and inner plexiform layer (GCIPL) and axonal retinal nerve fiber layer (RNFL). Their research aimed to evaluate the diagnostic value of both imaging techniques in cases of VFDs attributed to pituitary masses (32).

Furthermore, Rashid et al. employed OCT to detect alterations in macular ganglion cells and the inner plexiform layer (GCIPL) as well as the axonal retinal nerve fiber layer (RNFL). Their study sought to uncover the connections between these changes and the onset of VFDs. GCIPL and RNFL offer straightforward and dependable objective measurements, and are thus regarded as having predictive value for visual function (33).

Jeon et al. conducted a comparative analysis of retinal thickness measured using OCT and mass biometrics measured through MRI in patients experiencing temporal VFDs due to mass compression. Their findings indicated that, in cases of temporal VFDs caused by chiasmal compressive mass, MRI outperformed OCT in predicting postoperative VFDs recovery (34). Yoneoka et al. performed nasal endoscopic surgery on patients with visual symptoms attributed to pituitary tumors. During perioperative diagnostic MRI and ophthalmological assessments, they examined RNFL and ganglion cell complex thickness (GCC) using OCT as potential predictors of visual field prognosis (35).

Optical Coherence Tomography Angiography (OCTA) is a non-invasive blood flow detection technology developed as an extension of OCT. This advanced technique acquires clear fundus images by calculating variations in the movement of red blood cells within retinal blood vessels using computer algorithms. OCTA enables the precise visualization of lesions at different levels and locations within the eye, offering a detailed view of retinal vascular structures (36, 37). Agarwal et al. employed OCTA to assess morphological alterations in the posterior retinal choroidal vessels and compared these findings with results from clinical examinations and other imaging techniques. Their study revealed that OCTA can effectively depict changes in the microvascular structure of retinal vessels and serves as a sensitive tool for detecting HIV retinopathy (38).

#### 3.1.2 MRI and its associated functional imaging

Functional imaging has played a significant role in supporting the treatment of VFDs and certain associated surgical procedures. One such technique, MRI, utilizes magnetic fields and radio waves to generate a cross-sectional image of a patient’s body (39).

Beam imaging of the operating room (OR) has demonstrated its effectiveness in preventing VFDs when conducted within interventional MRI (iMRI) setups. Vakharia et al. observed that the implementation of OR tractography with overlay, conducted outside of an iMRI suite, can be effectively employed during approaches to the temporal horn of the lateral ventricle. However, it is worth noting that this approach carries a 5% risk of VFDs (40). David et al. conducted a study in which they conducted preoperative and postoperative MRI scans, including T1-MPRAGE and diffusion tensor imaging, along with motor field measurements in patients with temporal lobe epilepsy, following Goldmann criteria. Their goal was to elucidate the tissue-specific aspects of VFDs severity, employing analyses at both the voxel and network levels. Additionally, they explored the imaging correlations before and after amygdalotomy (41). Saeki comprehensively analyzed the distinct clinical presentations observed in patients with Rathke’s cleft cyst (RCC) using MRI models. Among these manifestations, VFDs emerged as one of the most significant. This exploration of the growth mechanism and treatment options for these cysts is pivotal for effectively monitoring and managing patients with RCC (42). Cho et al. put forth a hypothesis supporting the idea that the central 15° of the visual field corresponds to the posterior 25% of the visual cortex. They arrived at this conclusion by studying patients with reliable static perimetry that revealed homonymous hemianopias and employing high-resolution brain imaging. Their aim was to enhance the accuracy of lesion range assessment through MRI (43).

fMRI represents an innovative technology built upon the foundation of MRI. It is a neuroimaging technique that leverages magnetic resonance imaging to assess hemodynamic changes triggered by neuronal activity, primarily within the brain or spinal cord (44, 45). Diffusion Tensor Imaging (DTI), a novel approach for characterizing brain structure, is a specialized variant of MRI (46, 47). The progress in DTI and fMRI over the recent decades has significantly improved our comprehension of brain structure and function in patients with central nervous system diseases (48, 49).

In a study conducted by Cho et al., they attempted to establish a correlation between VFDs in patients with ischemic stroke and the location of lesions identified in MRI scans. However, their attempts to utilize MRI for assessing the progression of VFDs in patients yielded unsatisfactory results. The authors proposed that the limited effectiveness of their approach may be attributed to the subtle radiological characteristics of these lesions, making accurate radiological assessment either unattainable or exceeding the scope of the visual field testing protocol (50). Ying et al. conducted a study in which they administered structural imaging and resting-state fMRI scans both before and after surgery in patients with suprasellar tumors that presented with VFDs. Their objective was to investigate alterations in both the structure and function of the visual cortex at various time points before and after the decompression of the optic nerve (51).

Souza et al. conducted preoperative and postoperative DTI scans in epilepsy patients to assess changes in their visual field (52). James et al. employed DTI to track Meyer’s loop (ML) with the aim of preventing postoperative VFDs following anterior temporal lobectomy (ATL) for drug-resistant temporal lobe epilepsy (TLE) (53). Cui et al. assisted in anterior temporal lobectomy using DTI, optical radiation mapping, micro neuronavigation, and intraoperative magnetic resonance imaging to minimize the risk of VFDs (54). Souza et al., by combining DTI and Humphrey perimetry in patients, established correlations between post-TLE surgery Visual Radiation (OR) microstructure and VFDs, proposing that postoperative OR tractography following DTI could aid in VFDs evaluation (55). Yin et al. evaluated the incidence of VFDs by detecting radiation injury to the optic nerve through DTI in patients who underwent laser interstitial thermal therapy-amygdalohippocampectomy (LITT-AHE) for medically refractory mesial temporal lobe epilepsy (MTLE) (56). Quigg et al. utilized MRI to investigate the impact of volume changes on the Visual Field Defect Ratio (VFDR) and to comprehend the relationship between VFDR, epilepsy remission, and driving status 3 years after Stereotactic Radiosurgery (57). Tong et al. used optic nerve radiation-DTI during neuronavigational-guided surgery for occipital arteriovenous malformations, aiming to reduce the severity of postoperative VFDs and assess factors contributing to visual field preservation (58). Faust et al. conducted fiber tracking examinations using DTI on patients with tumors in the temporal lobe axis prior to surgery. After merging the DTI data with the corresponding magnetized fast gradient echo (MP-RAGE) images, they achieved tumor reconstruction and visualization of neural fibers. Their findings indicated that this approach is beneficial in reducing the likelihood of VFDs (59). Yogarajah et al. (60) employed diffusion tensor beam imaging to segment patients’ white matter and evaluate the risk of VFDs on an individual basis following anterior temporal lobectomy. Voets et al. (61) utilized DTI to assess the penetration of a laser catheter into the optical radiation and further complemented the evaluation of optic nerve injury. Lilja et al. (62) delved into the application of DTI as a method for the objective assessment of anterior visual pathway damage resulting from pituitary adenoma. They posited that DTI could offer objective data, identify early indicators of damage, and function as an additional diagnostic tool to help determine surgical indications for pituitary adenoma cases. Winston et al. (63) suggested that DTI can be harnessed to map the visual radiation and categorize the risk in patients before anterior temporal lobotomy (ATLR). This approach integrated bundle display into interventional MRI to facilitate guided surgery (63).

Diffusion Spectrum Imaging (DSI) is a recently developed MRI technique capable of mapping intricate fibrous structures in tissues with high angular resolution by imaging the spectroscopy of tissue water diffusion. This advanced method is particularly valuable for detecting visual pathways (64). Liang et al. examined the clinical utility of DSI for the quantitative assessment of visual pathway irregularities, with the aim of predicting the extent of VFDs in patients with pituitary adenoma. Their findings led to the conclusion that DSI can offer quantitative data to identify visual abnormalities and may serve as a potential diagnostic tool for assessing the degree of VFDs in pituitary adenoma cases (65).

Kim et al. uncovered that alterations in early resting-state functional connectivity (RSFC), as assessed by fMRI, could serve as a predictive factor for the recovery of VFDs in patients with acute stroke (66). Rutland et al. (67) harnessed 7-T diffusion-weighted MRI (DWI) for probability-based imaging of optic tracts and radiation to investigate microstructural damage caused by pituitary macroadenomas. The distinct dispersion characteristics of ultrahigh field 7-T DWI allowed for the characterization of optic tract injuries in patients experiencing cross-compression prior to surgery, offering valuable insights into the prognosis of vision recovery. In another study, van Lanen et al. (68) developed a quantitative scoring method to evaluate VFDs following anterior temporal lobectomy using static visual field tests. They evaluated the feasibility of this approach for clinical applications.

Furthermore, numerous other methods have gained widespread use in this field. Pruckner et al. adopted a multimodal approach to assess visual outcomes following epilepsy surgery. They presented clinical and neuroimaging evidence that suggested anterior temporal lobectomy (ATL) carried a greater risk and led to more severe postoperative VFDs compared to temporal selective amygdalohippocampectomy (tsSAHE) (69). Winston et al. reported that incorporating optic nerve radiography during anterior temporal lobectomy (ATLR) for refractory temporal lobe epilepsy (TLE) had the potential to reduce the severity of postoperative VFDs without impacting seizure outcomes or hippocampal resection (70).

On the whole, MRI has relatively low spatial resolution, especially when assessing microscopic structural changes in the visual pathway, compared to other imaging modalities like OCT. In one study, researchers found it difficult to assess the extent of brain lesions with conventional MRI, with 11.7% of cerebral palsy patients having normal conventional MRI findings (71). In contrast, OCT can provide higher spatial resolution, allowing detection and assessment of microscopic changes in visual pathway structures like thinning of the ganglion cell layer (GCL) and inner plexiform layer (IPL), and demonstrate visual field defects caused by brain lesions (72). However, it should be noted that MRI can provide valuable information about the extent of white matter tract injury, especially for assessing white matter damage (73). Moreover, compared to OCT, a unique advantage of MRI is its capability for comprehensive, three-dimensional analysis of brain structure and neural pathways.

With advances in MRI techniques such as high-field MRI, fMRI, and DTI, our understanding of the pathophysiology of VFDs have been further enhanced. High-field MRI utilizing stronger magnetic fields can provide higher signal-to-noise ratio and better resolution, which helps better image deep brain structures and visualize smaller structures more clearly. This allows more accurate observation of the anatomical and morphologic characteristics of various structures along the visual pathway, including the optic chiasm and lateral geniculate nuclei, by examining changes in these anatomical structures to gain deeper insight into the pathophysiological mechanisms of visual field defects (74). fMRI can monitor blood flow changes in active brain areas to understand functional maps and networks in the brain, which is critical for studying the function of visual processing regions and brain plasticity in VFDs patients (75). DTI is another specialized MRI technique that measures the diffusion properties of water molecules in brain tissue. Since water molecules diffuse more easily along the direction of neural fiber bundles, DTI can be used to map the white matter fiber tracts in the brain, including the visual radiations (76). DTI can also more precisely quantify and assess the integrity of vision-related nerve fibers using various diffusion coefficients like FA, l1, l23 and MD to predict whether visual field defects will occur (77).

### 3.2 Ophthalmic diseases

Eye diseases are indeed significant contributors to VFDs. The issue of excessive eye strain in modern society is becoming increasingly severe, leading to a rising incidence of various eye conditions. Among the most common are glaucoma, retinal damage, and various other visual problems that can result in VFDs. Regular eye check-ups and eye health practices are crucial for preventing and managing these conditions (78, 79). Numerous studies have made efforts to employ functional imaging techniques for investigating complications associated with VFDs in eye diseases, mainly OCT and its related functional imaging techniques.OCT is also a widely utilized tool for the detection of various eye diseases. For instance, Ohguro et al. used measurements of RNFL and VFDs, determined through OCT, to differentiate between cases of naso-optic hypoplasia (NOH) with NOH-like temporal VFDs, which is associated with NOH, and cases of glaucoma (80). Kallab et al. (81) conducted measurements to investigate the focal structure–function correlation of capillary density (CD). They utilized OCT to measure the structural thickness of the retinal nerve fiber layer around the optic disc (RNFL-T), in addition to employing OCT-angiography (OCT-A) or a combination of both techniques. Their aim was to compare VFDs in patients at various stages of glaucoma, ranging from early to late (81).

Spectral Domain Optical Coherence Tomography (SD-OCT) represents the second generation of OCT technology. When compared to the first-generation Time-Domain OCT technology, it boasts clear advantages in terms of imaging speed, signal-to-noise ratio, and sensitivity. As a result, SD-OCT plays a pivotal role in the fields of ophthalmic imaging and functional imaging (82, 83). As a result of its advantages, SD-OCT has seen increased usage in recent years. Miki et al. utilized Spectralis SD-OCT to detect the rate of RNFL loss in eyes with VFDs. Their findings suggest that measuring SD-OCT RNFL loss rates may serve as a valuable tool for identifying patients at a high risk of experiencing visual field loss (84). Wandji et al. employed SD-OCT to identify the presence of buried optic disc drusen (BODD). Additionally, they utilized Garway-Heath imaging to identify the associated VFDs. Their study focused on comparing the relationship between the presence of buried optic disc drusen and the resulting VFDs (85).

Various imaging techniques have been explored for assessing visual function and disorders. For instance, Kwon investigated the correlation between parameters related to the Foveal Avascular Zone (FAZ) and central vision function. This was achieved by measuring the FAZ area, circumference, roundness, and parafoveal vessel density using OCTA images. Such studies help to advance our understanding of the relationship between structural features of the eye and visual function (86). Carvalho et al. employed fMRI-based neural modeling to investigate hypothesized alterations in population receptive fields (pRFs) linked to VFDs in glaucoma. Their study revealed that local pRFs in the visual areas were associated with masking the VFDs, thereby making it challenging for patients to detect changes in their VFDs. This research sheds light on the neural mechanisms involved in VFDs perception and the impact of pRFs on visual awareness (87). In summary, the OCT series of functional imaging techniques have predominantly found applications in the realm of eye diseases.

The studies mentioned above have been compiled and summarized in Table 1 for reference and easy access. These investigations have contributed to our understanding of the various aspects of VFDs and their associations with different conditions, offering valuable insights for clinical diagnosis and management.Early diagnosis and accurate differentiation of ophthalmic diseases is the most important step in preventing and halting their onset and progression. Different imaging modalities have their own merits in early diagnosis of various diseases and their differentiation. OCT has demonstrated good performance in early glaucoma detection. By conducting a global region assessment using Cirrus OCT, a moderate consistency (*κ* = 0.420) was found between Cirrus OCT retinal nerve fiber layer thickness (RNFLT) measurements and neuroretinal rim area (RA) classifications. Additionally, by comparing results from Cirrus OCT between normal and early open-angle glaucoma groups, Cirrus OCT parameters were found to have good diagnostic ability for glaucoma with high sensitivity and specificity (88). MRI can detect early brain changes in glaucoma, showing microstructural abnormalities in the visual pathway and changes and atrophy in the central visual system, providing important information for early diagnosis and helping diagnose and monitor the progression of glaucomatous damage (89). For diabetic retinopathy, OCT can detect characteristic changes associated with early diabetic retinopathy such as macular edema, which is crucial for discovering diabetic retinopathy early on (90). MRI can serve as a potential screening method and possible quantitative physiological biomarker for early signs of diabetic retinopathy in type 2 diabetes patients. Studies show MRI can provide quantitative information on the early status of diabetic retinopathy by measuring retinal oxygenation levels (91). OCT can also aid in diagnosing early age-related macular degeneration (AMD) lesions by observing thickness and morphological changes in the macular area. Additionally, OCT image parameters are associated with the degree and progression of AMD lesions (92). MRI has shown some limitations in detecting early AMD lesions. Specifically, early AMD did not show significant associations with brain MRI changes like white matter lesions (WML), subcortical white matter volume (SW) and ventricular volume (VE), so MRI is less utilized for early detection of AMD (93).

**Table 1:** Summaries of the studies.

Disease classification	Study group	Functional imaging method	Detection content	Conclusions	Study
Neurological disease	Patients with a pituitary mass	OCT + MRI	OCT: GCIPL+RNFL MRI: Height and size of the mass, the displacement of optic chiasm, and the direction of mass extension	OCT and MRI can diagnose VFDs	Suh et al. (32)
	Patients with acute occipital stroke	OCT	GCIPL and RNFL	GCIPL and RNFL can predict visual function	Rashid et al. (33)
	Patients with pituitary adenomas	DSI + MRI	Optic chiasmal compression and VFD	DSI can diagnose the degree of VFDs in pituitary adenomas	Liang et al. (65)
	Patients with suprasellar tumor	resting-state fMRI	GMV in the bilateral pericalcarine cortex	The postoperative visual improvement is reflected in the increased GMV and ALFF	Ying et al.
	Patients with therapy-refractory temporal lobe epilepsy	MRI (T1-MPRAGE and DTI)	Whole-brain gray matter (GM) and white matter (WM)	Neither in the GM, WM, nor in network metrics we found preoperative correlates of VFD severity.	David et al. (51)
	Patients with temporal VFD caused by chiasm compression disorder	OCT + MRI	OCT: retinal thickness MRI: mass biometrics	MRI can better predict postoperative VFD recovery in patients with temporal VFD caused by chiasm compression disorder	Jeon et al. (34)
	Patients with occipital arteriovenous malformations	DTI	optic radiation was displayed during neuronavigation surgery	DTI navigation facilitate surgery and preserve the patient’s visual field	Tong et al. (58)
	Patients with intraaxial tumors of the temporal lobe	DTI	tumor reconstruction and fiber visualization	Preoperative visualization of the OR may help in avoiding postoperative VFDs	Faust and Vajkoczy (59)
	Patients who do anterior temporal lobe resection (ATLR)	Diffusion tensor tractography	distance from the tip of Meyer’s loop to the temporal pole and the size of resection	Diffusion tensor tractography of the optic radiation is useful to assess the risk of postoperative VFDs following anterior temporal lobe resection.	Yogarajah et al. (60)
	Patients who developed persistent VFDs after Selective laser amygdalohippocampotomy	Diffusion tractography	laser catheter penetration of the optic radiations	The incidence of VFDs after sah is lower than that of open temporal lobe surgery	Voets et al. (61)
	Patients with pituitary adenomas	DTI	chiasmal lift, VFD, and DTI parameters from the optic tracts	DTI can detect pathology and degree of injury in the anterior visual pathways that were compressed by pituitary adenomas	Lilja et al. (62)
	Patients undergoing ATLR	DTI	Tractography of the optic radiation	Dissemination of preoperative tractography to neurosurgery may reduce the risk of VFD	Winston et al. (63)
	Patients who were diagnosed with (hippocampal sclerosi) HS and underwent temporopolar amygdalohippocampectomy (TP-AH)	DTI	inferior frontooccipital fasciculus and optic radiation tractography	TP-AH surgery can reduce the incidence of VFD	de Souza et al. (52)
	Patients with and VFD following ATL	DTIT	Meyer’s loop (ML) tracking	DTIT can predict the occurrence of postoperative VFDs	James et al. (53)	
	Patients with medically refractory temporal lobe epilepsy undergoing ATLR	DTI + iMRI	the size of ATLR and The optic radiation	The combination of optic radiation mapping, microscopic-based neuronavigation and iMRI aided can reduce the risk of VFDs in ATLR	Cui et al. (54)
	Patients who underwent temporal lobe epilepsy (TLE) surgery	DTI	perimetry and optic radiations tractography	DTI postoperative OR tractography help evaluating VFD	de Souza et al. (55)
	Patients who underwent LITT-AHE for medically refractory MTLE	DTI	postprocedural visual field testing and optic radiation fiber tracts	Incorporating OR mapping through DTI help reduce the risk of VFD following laser ablation AHE	Yin et al. (56)
	Patients with unilateral hippocampal sclerosis	MRI	Hippocampal volume changes	The nature of VFD was consistent with lesions of the optic radiations	de Souza et al. (55)
	Patients with VFDs due to pituitary tumors underwent endoscopic endonasal surgery	MRI + OCT	OCT: RNFL and thickness of ganglion cell complex (GCC)	Early decompression is crucial for VFDs recovery	Yoneoka et al. (35)
	Patient with pituitary macroadenomas	DWI	Optic tracts and radiations	Diffusion characteristics enabled by ultra-high-field DWI allow preoperative assessment of the prognosis of VFDs	Rutland et al. (67)
	Patients with drug-resistant TLE who underwent ATL	MRI	Temporal lobe resection length	A new quantitative scoring method for the assessment of postoperative VFDs after temporal lobe epilepsy surgery	van Lanen et al. (68)
Ophthalmic diseases	Primary open angle glaucoma patients	OCT + OCTA	OCT: peripapillary retinal nerve fiber layer thickness (RNFLT) OCTA: capillary density (CD)	The combined structure-functional correlation of RNFLT and CD was better than thickness or vascular information.	Kallab et al. (81)
	Glaucoma suspects	SD-OCT	RNFLT	RNFL loss measured by SD-OCT can be identified to predict the occurrence of VFDs	Miki et al. (84)
	Patients diagnosed with buried optic disc drusen (BODD)	OCT	Macular and papillary	correspondence between BODD location, RNFL damage, and VFD distributions	Nana Wandji et al. (85)
	Patients with glaucoma with peripheral VFD and central VFD	OCT	FAZ area, perimeter, and circularity and parafoveal vessel density	FAZ perimeter can be used to identify glaucoma patients with CVFDs	Kwon et al. (86)
	Glaucoma patients	fMRI	fMRI-signals and population receptive field (pRF)	pRF can prevent patients from noticing the VFDs	Carvalho et al. (87)

### 3.3 Bibliometrics analysis

We retrieved studies published between January 1, 1999, and December 31, 2023, from Web of science core collection and employed a combined quantitative and qualitative approach to data analysis. Furthermore, we conducted a comprehensive search of the entire literature. The data was then structured to identify the keywords used in the literature based on their frequency and timeline. This helps identify current research hotspots and aids in predicting future trends in the field of functional imaging. However, the conclusions drawn from bibliometric analysis depend on the quantity and quality of the considered literature. Also, as research outcomes in some fields might manifest through alternative mediums like patents, bibliometric analysis may not comprehensively represent the entire discipline. Therefore, as a macroscopic analytical method, while bibliometric analysis has its limitations, it also offers unique insights into the development of functional imaging research.

**Figure 1:**
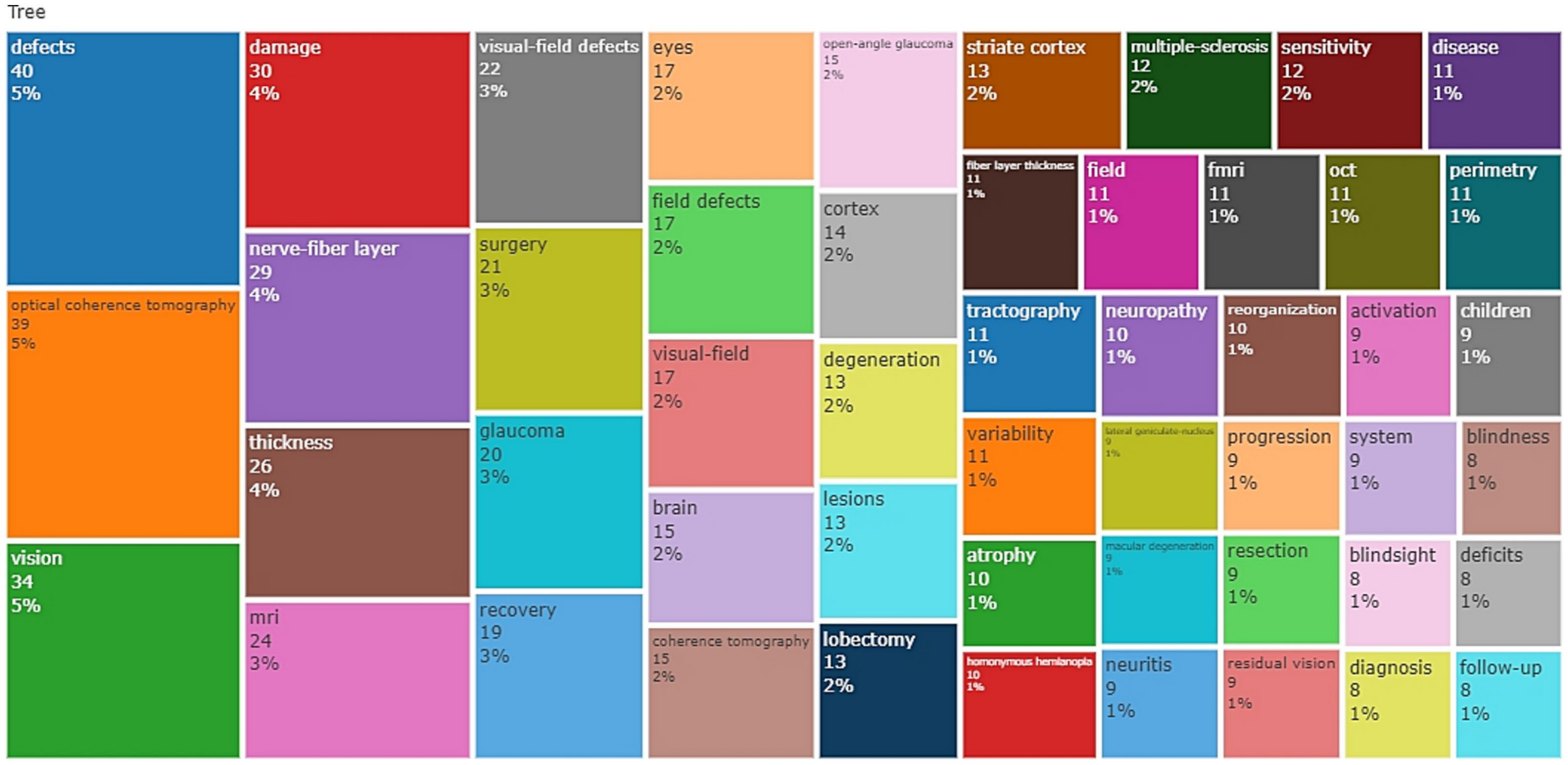
Treemap of the keywords, this figure presents the word frequency analysis of keywords in related fields.

The results of the bibliometric analysis (94–96), as depicted in Figure 1, indicate that OCT is the most widely used and popular functional imaging method, accounting for the highest percentage among all keyword searches, with a 5% share. Following OCT, the MRI series of technologies, with “MRI” as a keyword accounting for 3%, and “fMRI” as a keyword accounting for 1%, represent the second most commonly used group of functional imaging methods.

Moreover, the analysis of keywords not only reflects the application of different functional imaging techniques but also provides insights into the conditions causing VFDs and the utilization of imaging technologies. In alignment with our prior detailed literature analysis, the appearance of the keyword “Surgery” with a frequency of 3% suggests that functional imaging techniques are more frequently employed in surgical contexts. The frequency of the keyword “Glaucoma” also reached 3%, indicating a higher occurrence of VFDs caused by glaucoma and a greater need for the application of functional imaging technology.

Furthermore, a significant number of keywords related to the brain and the nervous system appeared, reinforcing our earlier classification of diseases into neurological diseases and eye diseases. This suggests that functional imaging techniques are frequently utilized in both of these disease categories.

**Figure 2:**
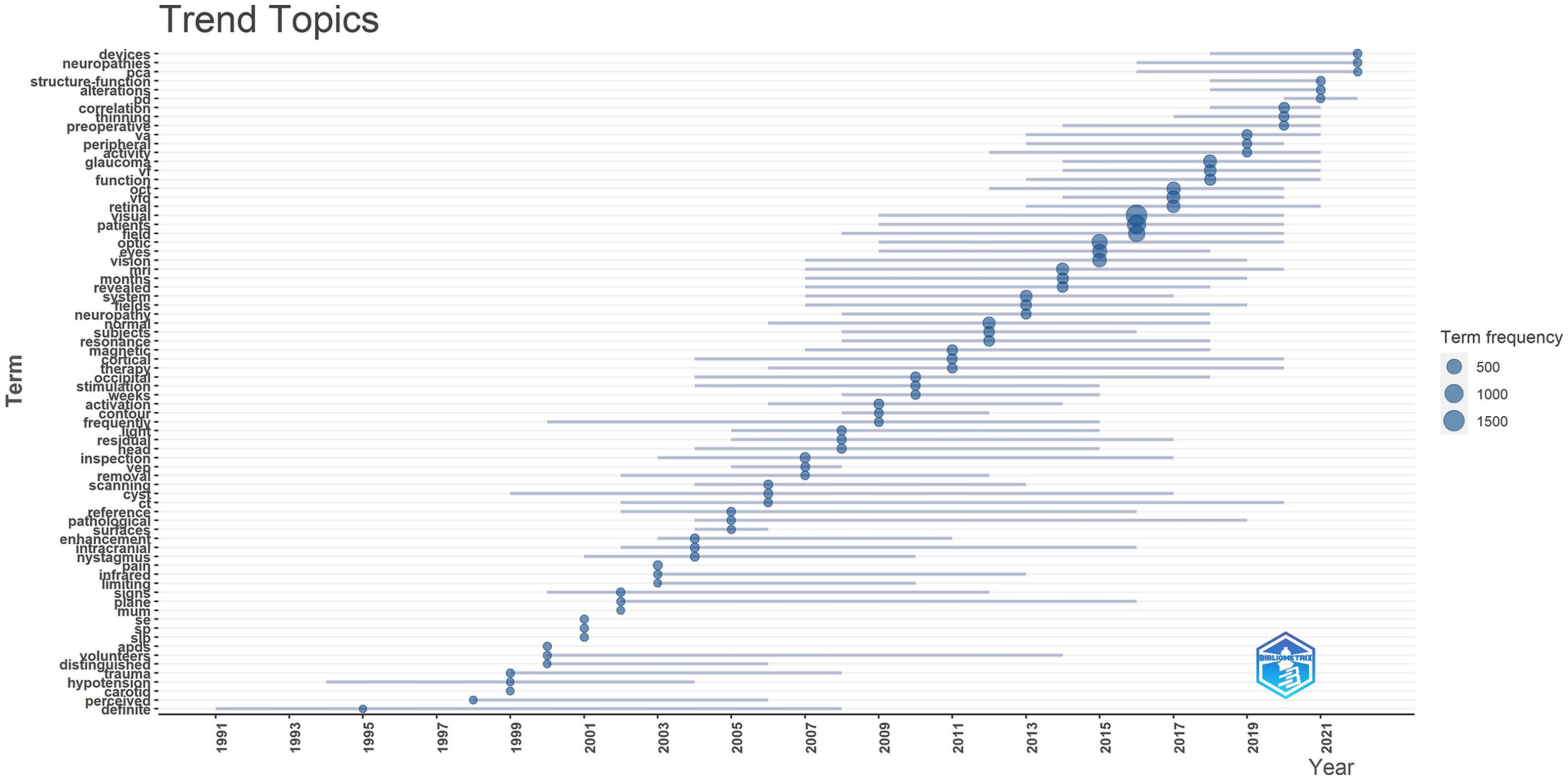
Trend topics, this figure shows the hot spot changes of keywords in related fields over time.

Figure 2 reveals that in recent years, there has been significant interest in conditions such as dysthyroid optic neuropathy, idiopathic intracranial hypertension, and nerve sheath meningioma. These conditions have captured considerable attention and research focus within the field of functional imaging.

Furthermore, the data highlights that OCTA is among the most advanced and cutting-edge technologies currently in use. This indicates that OCTA is at the forefront of functional imagi ng and has garnered substantial interest and development in recent years.

## 4 Discussion

With the continuous advancement of science and technology, the realm of functional imaging technology is constantly evolving and expanding. These innovative techniques are finding increasing utility in the realm of VFDs disease. Following a comprehensive review of existing clinical studies, we conducted a macro-economic analysis of this field, revealing that OCT stands as the most commonly employed tool, closely followed by MRI. Among the various MRI-related tools, fMRI is widely embraced, while OCTA emerges as the cutting-edge and state-of-the-art technology within the OCT domain.

OCT is mainly used for disease assessment, and DTI and MRI series are often used to assist surgical operations and evaluate the progression of patients’ conditions. With the continuous advancement of MRI technology, especially the introduction of high-field MRI, the understanding of the pathophysiology of visual field defects has been enhanced. High-field MRI refers to the use of magnetic resonance imaging devices with higher magnetic field strength (97, 98). The advantage of this technology is to improve the signal-to-noise ratio and contrast of images, allowing doctors to distinguish subtle structural changes more clearly. In assessing the microstructure of the visual pathway, high-field MRI can more accurately reveal details of neurons, fiber bundles, and other microstructures. In addition, high-field MRI can provide richer functional information, such as quantitative measurements of brain activity and metabolism, which are crucial for understanding the pathogenesis and pathophysiological changes of visual field defects (98–100). As one of the high-field MRI technologies, DTI focuses on the directional diffusion of water molecules in tissues, providing detailed quantitative information about neural fiber bundles, revealing the degree and location of damage to neural fiber bundles (101, 102). Application of DTI during epileptic surgery or other vision-related surgeries helps reduce the risk of postoperative visual field defects (103). Compared to MRI, DTI may have higher sensitivity and specificity in assessing abnormalities in visual function. It has been widely used to study vision-related diseases such as retinitis pigmentosa, glaucoma, etc. Results demonstrate that DTI can detect structural and functional abnormalities in the optical pathway, further assisting diagnosis and assessment of disease progression (104). MRI, with relatively high spatial resolution, can provide detailed information and high-resolution images of tissue structure and organ morphology from the perspective of overall anatomy (105, 106). Together, they help surgeons more accurately locate and protect important visual pathway structures during surgical interventions, reduce surgical risks, and provide important evidence for personalized treatment plans.

In recent years, OCT-related technologies, especially the second-generation SD-OCT, have improved scanning speed, enhanced imaging resolution and depth, and integrated more closely with artificial intelligence, achieving significant progress in addressing VFDs caused by ophthalmic diseases (107–109). Another important technology, OCTA, has certain advantages in detecting early changes in VFDs. Traditional OCT mainly generates images by measuring the reflectivity of light, primarily reflecting structural information of tissues. In contrast, OCTA generates images by detecting blood flow signals, providing more detailed information about vascular structures (110). This allows OCTA to detect early changes more sensitively in VFDs, such as changes in local blood flow, microcirculation disorders, and changes in vascular density, which may be difficult to observe in traditional OCT images. Additionally, OCTA can provide information on the three-dimensional vascular structure and differentiate vascular distribution at different depths through scans, aiding in more accurate localization of VFD lesions (36, 111–113). However, OCTA also has some limitations and challenges. For example, it is highly dependent on the flow rate, and signal loss may occur in low perfusion states or low blood flow velocities. Additionally, OCTA has limitations in resolution and depth, restricting its application in certain situations (36, 114).

Overall, these technologies almost cover all aspects of the patient treatment process, playing important clinical roles in diagnosis, treatment, and prognosis. They significantly strengthen patient care, making monitoring and assessing unforeseen risks possible. Regarding specific diseases, literature analysis suggests that functional imaging techniques may see greater adoption in surgical contexts, with a growing demand for their application in the domain of glaucoma. Recent attention has also been directed towards disorders such as dysthyroid optic neuropathy, idiopathic intracranial hypertension, and nerve sheath meningiomas. These conditions have garnered substantial interest and research focus within the functional imaging field.

## 5 Conclusion

In conclusion, functional imaging techniques such as DTI, OCT, and MRI have become invaluable tools in the diagnosis, treatment, and management of visual field defects. Each modality provides unique insights, yet also possesses its own limitations and considerations. As the field continues to progress, the integration of functional imaging into clinical practice will further enhance our understanding and care of patients with VFDs. Nevertheless, additional research is warranted to optimize and standardize protocols across institutions. Overall, this review highlights the vital role of functional imaging in advancing the care of visual field defects through refined characterization of structure–function relationships.

## Author contributions

WC: Writing – original draft. JL: Writing – original draft. TJ: Writing – original draft. ML: Writing – review & editing.
